# Biogenic volatile organic compound emission characteristics of dominant tree species in temperate broad-leaved Korean pine forests in Northeast China

**DOI:** 10.3389/fpls.2026.1858171

**Published:** 2026-06-19

**Authors:** Dingyi Pei, Anzhi Wang, Jiabing Wu

**Affiliations:** 1 CAS Key Laboratory of Forest Ecology and Silviculture, Institute of Applied Ecology, Chinese Academy of Sciences, Shenyang, China; 2 University of Chinese Academy of Sciences, Beijing, China

**Keywords:** biogenic volatile organic compounds, emission rate, isoprene, monoterpene, temperate broad-leaved Korean pine forest

## Abstract

Biogenic volatile organic compounds (BVOCs) play important roles in atmospheric chemistry, ozone formation, and secondary organic aerosol production. However, field-based measurements of species-specific BVOC emissions and emission factors remain scarce for temperate broad-leaved Korean pine forests in Northeast China. In this study, BVOC emissions from five dominant tree species (*Pinus koraiensis*, *Quercus mongolica*, *Tilia amurensis*, *Acer mono*, and *Fraxinus mandshurica*) were quantified using *in situ* dynamic headspace sampling coupled with gas chromatography-mass spectrometry (GC-MS). Seasonal variation patterns, diurnal dynamics, compound composition, and environmental responses were analyzed. Remarkable interspecific differences in BVOC composition and emission rates were observed. *Pinus koraiensis* was dominated by monoterpene emissions, whereas broad-leaved species were primarily dominated by isoprene. *Quercus mongolica* exhibited the highest isoprene proportion (93.6%) and the highest overall emission rates among the investigated species. Monoterpene composition also differed substantially among tree species. BVOC emissions exhibited pronounced seasonal and diurnal variation patterns, with emission rates generally peaking during summer and around midday. Temperature exerted stronger effects on BVOC emissions than photosynthetically active radiation (PAR) across most species. This study provides field-based species-specific BVOC emission data and standardized emission factors for dominant tree species in temperate broad-leaved Korean pine forests of Northeast China. The results improve the understanding of BVOC emission patterns in temperate forest ecosystems and may help reduce uncertainties in regional BVOC emission inventories and atmospheric chemistry modeling.

## 1 Introduction

In forest ecosystems, biogenic volatile organic compounds (BVOCs) released by plants serve as an important link connecting plant physiological processes and atmospheric chemical processes, and exert significant impacts on the formation of regional ozone (O_3_) and secondary organic aerosols (SOA) (Fitzky et al., 2019; Zhang et al., 2022). BVOCs mainly include isoprene, monoterpenes, sesquiterpenes, carbonyl compounds, a series of alcohols, oxides and esters (Sartelet et al., 2012; Lun et al., 2020), They represent the major source of global non-methane volatile organic compounds (Helmig et al., 2007; Loreto and Schnitzler, 2010; Guenther et al., 2012), with annual emission of approximately one billion tons, among which isoprene and monoterpenes account for the most two large proportions respectively (Guenther et al., 2020). Isoprene and monoterpenes in plant BVOCs are primarily biosynthesized via the plastidial 1-deoxy-D-xylulose 5-phosphate/2-C-methyl-D-erythritol 4-phosphate (DOXP/MEP) pathway, whereas sesquiterpenes are mainly produced through the cytosolic mevalonic acid (MVA) pathway (Ghimire et al., 2022; Cheng et al., 2025; Yang et al., 2025). Differences in enzyme activities and substrate supply among different species lead to significant variations in their emission chemical compositions (Calfapietra et al., 2013; Li et al., 2021) and emission intensities (Fitzky et al., 2019; Zhang et al., 2022).

Generally, differences in metabolic pathways (Sharkey and Yeh, 2001), leaf structure (Duan et al., 2023) and volatile storage mechanisms among plant species (Loreto and Schnitzler, 2010), are the primary drivers of species-specific variations in BVOC emissions (Brosset and Blande, 2022). For instance, isoprene and monoterpenes dominate BVOC emissions in most temperate tree species, although their relative proportions vary considerably by taxon. Many deciduous broad-leaved trees (e.g., oaks) are typically strong isoprene emitters, whereas coniferous species (e.g., pine) predominantly release monoterpenes (Guenther et al., 1995; Sharkey and Yeh, 2001; Luo et al., 2025). Beyond inherent plant traits, BVOC emissions are also regulated by environmental factors including light (Monson and Fall, 1989; Guenther et al., 1995; Wang et al., 2022), leaf temperature (Sharkey et al., 2007; Monson et al., 2012), CO_2_ concentration, water availability, and nutrient status (Hao et al., 2025), leading to distinct diurnal and seasonal emission patterns.

Observations in European temperate forests have shown that broad-leaved tree species represented by European beech exhibit high isoprene emission characteristics, whereas coniferous species such as fir and spruce are dominated by monoterpene emissions, with emission rates strongly controlled by local temperature and light intensity (Laffineur et al., 2011; Dumont et al., 2026). These findings suggest that the composition and magnitude of BVOC emissions in different forest stands are jointly regulated by plant species, as well as climatic and *in-situ* environmental conditions.

In recent years, considerable progress has been made in the study of BVOC emissions. For example, by measuring BVOCs from 24 dominant forest tree species using dynamic headspace sampling coupled with gas chromatography-mass spectrometry, scientists demonstrated that isoprene and monoterpenes were the primary BVOC components emitted by forest trees, with significant differences in emission composition and magnitude between broad-leaved and coniferous species (Jing et al., 2020). Wu et al. found that ignoring seasonal variations would lead to large biases in BVOC emission estimates, highlighting the importance of seasonally dynamic emission factors for temperate tree species (Wu et al., 2024). A review on temperature effects on BVOC emissions identified temperature as a key environmental driver, which is closely linked to photosynthetic capacity and leaf thermoregulation, providing a theoretical basis for understanding the environmental regulatory mechanisms of BVOC emissions (Yang et al., 2025).

Broad-leaved Korean pine mixed forests represent a typical temperate forest ecosystem in Northeast Asia, characterized by diverse tree species composition and unique community structure. They play a vital role in regional carbon cycling, atmospheric chemical processes, and the regulation of atmospheric environment. Nevertheless, systematic observational data on BVOC emissions from dominant tree species in this region remain relatively scarce. In particular, long-term and comprehensive *in-situ* measurements are lacking regarding species-specific compositional differences, emission magnitudes, and their spatiotemporal dynamics. Most existing studies have focused on global-scale assessments or temperate forests in Europe and North America, whereas the local emission characteristics of temperate forests in Northeast Asia have not been fully quantified.

This study selected five dominant tree species in the broad-leaved Korean pine mixed forest of Northeast China—*Pinus koraiensi*, *Quercus mongolica*, *Tilia amurensis*, *Acer mono*, and *Fraxinus mandshurica*—as research objects. Using dynamic headspace sampling coupled with gas chromatography-mass spectrometry (GC-MS), the chemical composition and emission rates of BVOCs from each tree species were systematically measured, and their diurnal and seasonal variation characteristics were analyzed. Furthermore, the standard emission factors were calculated based on the *in-situ* measured data, which provides key parameter support for the construction of regional BVOC emission inventories and atmospheric chemical models of the broad-leaved Korean pine mixed forest in Northeast China. This work aims to further promote the understanding of species-specific BVOC emissions from temperate forests and their environmental driving mechanisms.

## 2 Study area and methods

### 2.1 Study area

This study was conducted in a temperate broad-leaved Korean pine forest located in the Changbai Mountains of Northeast China (42.40° N, 128.10° E; elevation: 801.5 m). The forest represents a typical climax mixed forest ecosystem in Northeast Asia. The study area is characterized by a temperate continental monsoon climate, with a mean annual air temperature of 3.5 °C and annual precipitation ranging from 600 to 800 mm, most of which occurs from June to August. The frost-free period lasts approximately 100–120 days, and the mean annual relative humidity is 71%-72%. The forest exhibits a typical multi-layered uneven-aged structure. The overstory is dominated by old-growth trees approximately 250 years old, with an average canopy height of 27 m and canopy cover of approximately 0.8. Understory shrub coverage ranges from 40% to 50%. The tree layer contains 15 woody species, among which *Pinus koraiensis*, *Quercus mongolica*, *Tilia amurensis*, *Acer mono*, and *Fraxinus mandshurica* are the dominant canopy species investigated in this study (Yu et al., 2011).

### 2.2 Sampling methods

**Figure 1:**
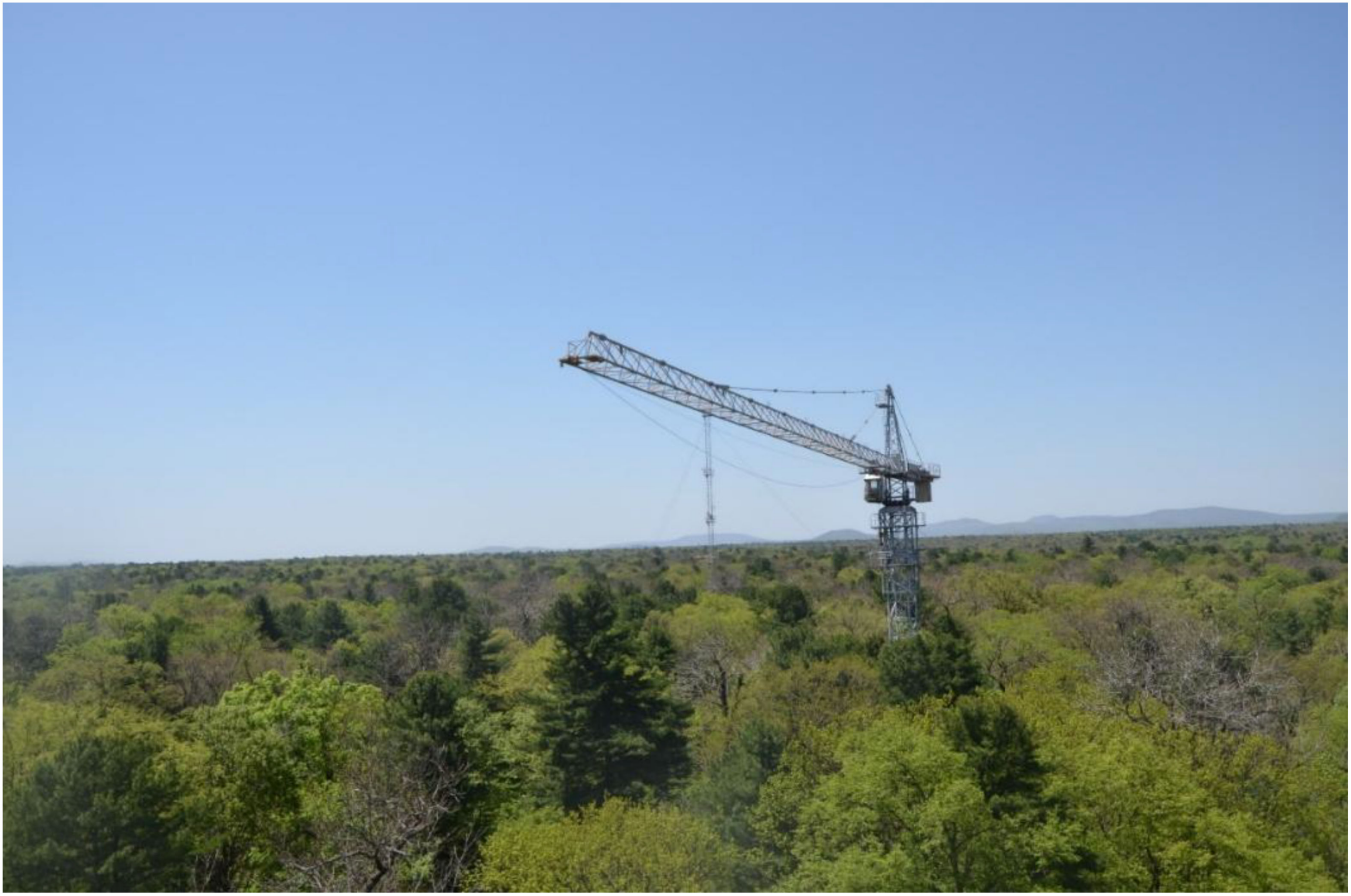
Photo of the tower crane at the Changbai Mountain forest experiment site.

Field sampling was conducted from May to October during 2023-2025, with measurements performed around the middle of each month. According to local phenological conditions, May, June-August, and September-October were classified as spring, summer, and autumn, respectively. Because most broad-leaved species had entered leaf senescence by mid-October, October measurements were only performed for the evergreen conifer *Pinus koraiensis*. BVOC samples were collected on sunny and low-wind days between 06:00 and 18:00 using a canopy crane system installed in the No. 1 permanent plot at the Changbai Mountain forest research site. Sampling branches were located within the upper canopy layer (approximately 20–27 m above ground) and selected from sun-exposed and leeward canopy positions (**Figure 1**).

Because canopy level BVOC measurements required the combined use of a crane platform and dynamic headspace enclosure system, dominant tree species were sampled sequentially rather than simultaneously. To minimize environmental heterogeneity among species, measurements were generally completed under comparable meteorological conditions and within the same observation day whenever possible. The sampling sequence and observation period were maintained as consistently as possible throughout the field campaigns, although minor adjustments were occasionally necessary due to weather conditions and canopy accessibility. For each tree species, three biological replicates were collected from branches with similar canopy positions and light conditions.

**Figure 2:**
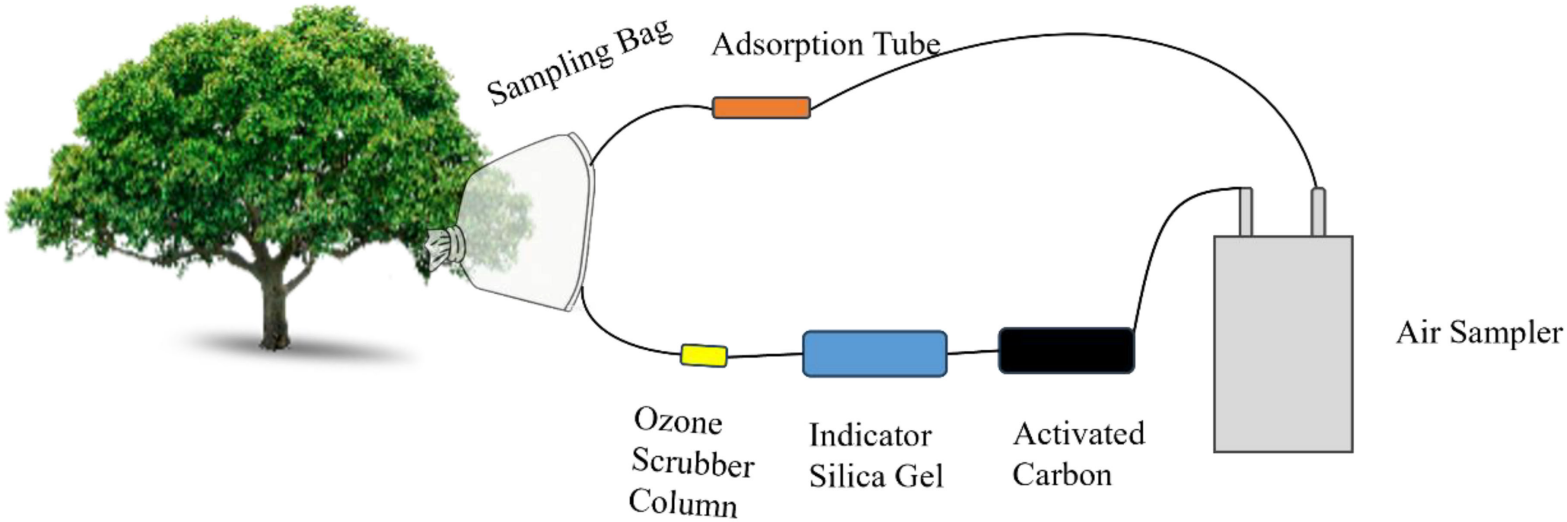
Dynamic headspace sampling process.

BVOC collection was conducted using a dynamic headspace sampling method (Liu et al., 2022; Ortega and Helmig, 2008). Healthy branches were enclosed in 15 L PTFE sampling bags connected through PTFE tubing to a circulation system equipped with a sampling pump, drying tubes containing activated carbon and color-indicating silica gel, and an ozone scrubber (**Figure 2**). Prior to formal sampling, the enclosure system was allowed to equilibrate for 30 min without connecting the adsorbent tube. During this pre-circulation period, purified air was continuously circulated through the enclosure at a flow rate of 300 mL min^−1^ to stabilize the internal microenvironment and reduce potential background contamination. After equilibration, a Tenax-TA adsorbent tube was connected to the outlet port of the sampling bag, and BVOCs were collected at a flow rate of 100 mL min^−1^ for 10 min (Tholl et al., 2006). Blank controls were simultaneously conducted using empty sampling bags without branches or leaves. The adsorbent tube was connected at the outlet side of the enclosure system with identical airflow conditions and sampling, and the resulting blank values were used for background correction during BVOC emission rate calculations.

After sampling, adsorbent tubes were immediately sealed in bags containing activated carbon and stored at 4 °C prior to analysis. Leaves enclosed during sampling were subsequently excised for leaf area determination and then dried to constant weight for biomass measurements (Niinemets et al., 2011).

The sampling bags were constructed from PTFE film and equipped with inlet and outlet ports fitted with check valves. Air circulation within the enclosure system was maintained using atmospheric air samplers, and BVOCs were enriched using stainless-steel adsorbent tubes (6 mm × 150 mm) packed with Tenax-TA. Prior to field sampling, activated carbon used in the drying tubes was heated at 160 °C for more than 5 h to remove residual volatile contaminants, then loaded into the gas drying tubes and sealed for natural cooling. Adsorbent tubes were thermally activated at 360 °C for 60 min under high-purity helium (≥99.999%) at a flow rate of 10 mL min^−1^. After activation, the tubes were also immediately sealed with airtight caps to prevent contamination and allowed to cool naturally to room temperature. The tubes were subsequently placed in sealed bags containing activated carbon and stored at 4 °C until field sampling (Niinemets et al., 2011).

### 2.3 Analytical methods

Collected BVOC samples were analyzed using thermal desorption coupled with gas chromatography-mass spectrometry (TD-GC-MS) (Li et al., 2021). Thermal desorption was performed using an Auto TDS-I thermal desorber (Beijing Tashideyan Instrument Co., Ltd., China) coupled with an ISQ LT gas chromatography-mass spectrometer (Thermo Fisher Scientific, USA). Mass spectral data acquisition and processing were conducted using Xcalibur software. Adsorbent tubes were thermally desorbed at 260 °C for 20 min to release absorbed organic compounds for GC-MS analysis. BVOC separation was performed using a DB-5MS capillary column (30 m × 0.25 mm × 0.25 μm). Helium was used as the carrier gas at a constant flow rate of 1.2 mL min^−1^. The GC oven temperature program was as follows: the initial temperature was maintained at 40 °C for 10 min, increased to 200 °C at a rate of 10 °C min^−1^, and then held for 5 min. The mass spectrometer was operated in electron ionization (EI) mode at 70 eV with a scan range of 40–210 amu.

Compound identification was performed by comparing mass spectra with the NIST mass spectral library combined with retention-time information of authentic standards. Quantitative analysis was conducted for 14 target BVOC compounds, including isoprene, methyl acetate, benzene, toluene, α-pinene, β-pinene, camphene, 3-carene, α-terpinene, terpinolene, limonene, myrcene, p-cymene, and ocimene.

Standard solutions at multiple concentration levels (0.025-0.5 μg mL^−1^) were prepared in methanol from a mixed standard solution containing all target compounds. Calibration curves were established using five concentration levels, with one analysis performed at each concentration level. Prior to analysis, the standard solutions were loaded onto conditioned Tenax-TA adsorbent tubes and analyzed using the same TD-GC-MS procedure as that applied to field samples. External calibration curves were generated based on peak areas obtained at different concentration levels for each target compound. All calibration curves showed strong linear relationships, with correlation coefficients (R²) greater than 0.99. Concentrations of BVOC compounds in the samples were quantified using the corresponding external calibration curves. Blank correction was applied to all quantified compounds to minimize potential background contamination from ambient air and the sampling system. Standard retention times, LODs, and LOQs of the target compounds are listed in **Table 1**.

**Table 1:** Chemical information of the 14 target VOCs.

Compound	CAS	Retention time	LOD (ng/mL)	LOQ (ng/mL)
Isoprene	78-79-5	2.69	38.90	117.88
Methyl acetate	79-20-9	3.78	20.87	63.23
Benzene	71-43-2	7.76	37.25	112.87
Toluene	108-88-3	13.78	18.50	56.05
α-Pinene	80-56-8	18.25	19.88	60.25
Camphene	79-92-5	18.69	16.79	50.86
β-Pinene	127-91-3	19.33	17.27	52.34
Myrcene	123-35-3	19.48	7.86	23.83
3-Carene	13466-78-9	19.94	8.04	24.37
α-Terpinene	99-86-5	20.12	9.25	28.04
Limonene	138-86-3	20.33	7.48	22.67
p-Cymene	99-87-6	20.42	6.47	19.61
Ocimene	13877-91-3	20.63	2.46	7.46
Terpinolene	99-85-4	20.88	8.29	25.13

### 2.4 Meteorological data

Meteorological data were obtained from long-term observation equipment mounted on an eddy flux tower. Among them, the conventional meteorological system provided photosynthetically active radiation (PAR) and air temperature (T) data. Data were collected and stored online using a data logger (CR1000X, Campbell Scientific, USA).

### 2.5 Calculation formulas

BVOC emission rates were calculated based on compound concentrations collected in the adsorbent tubes, sampling flow rate, and dry leaf biomass enclosed within the sampling bag according to the following equation:

(1) $$\epsilon =\frac{\left({\text{C}}_{\text{out}}-{\text{C}}_{\text{in}}\right)\cdot \text{Q}}{\text{m}}$$

Where, ϵ: BVOCs emission rate, μg·g^−1^·h^−1^; C_out_: BVOCs concentration at the sampling outlet, μg·mL^−1^; C_in_: BVOCs concentration in blank samples, μg·mL^−1^; Q: sampling flow rate, mL·h^−1^; m: leaf dry weight, g.

Emission rates calculated using **Equation 1** represent values under ambient field conditions during sampling. Standardized emission factors were subsequently estimated using the Guenther 1993 (G93) algorithm (Guenther et al., 1993). For isoprene, standardized emission rates were calculated as (**Equation 2**):

(2) $$\epsilon_{\text{ISOP}}=\frac{\epsilon }{{\text{C}}_{\text{L}}\cdot {\text{C}}_{\text{T}}}$$

Where, ϵ_ISOP_: isoprene emission rate under standard conditions (T_leaf_ =303K, PAR = 1000 μmol·m^-2^·s^-1^), μg·g^−1^·h^−1^; C_L_: light correction factor; C_T_: temperature correction factor.

For monoterpenes and other BVOCs, standardized emission rates were calculated as (**Equation 3**):

(3) $$\epsilon_{\text{MTs, OVOCs}}=\frac{\epsilon }{\exp[\beta \cdot (\text{T}-{\text{T}}_{\text{s}})]}$$

Where, ϵ_MTs, OVOCs_: emission rate of monoterpenes and other VOCs under standard conditions (T = 303K); β: empirical coefficient, taken as 0.10 K^−1^ for monoterpenes and other BVOCs, and 0.17 K^−1^ for sesquiterpenes; T: leaf temperature, K; Ts: leaf temperature under standard conditions, 303 K.

The light correction factor for isoprene was calculated as follows (**Equation 4**):

(4) $${\text{C}}_{\text{L}}=\frac{\alpha \cdot {\text{C}}_{\text{L}1}\cdot \text{L}}{\sqrt{1+\alpha^{2}\cdot {\text{L}}^{2}}}$$

Where, α: empirical coefficient, generally taken as 0.0027; C_L1_: empirical coefficient, generally taken as 1.066; L: photosynthetically active radiation flux, μmol·m^−^²·s^−1^.

The temperature correction factor for isoprene was calculated as follows (**Equation 5**):

(5) $${\text{C}}_{\text{T}}=\frac{\exp\frac{{\text{C}}_{\text{T}1}\cdot (\text{T}-{\text{T}}_{\text{s}})}{\text{R}\cdot {\text{T}}_{\text{s}}\cdot \text{T}}}{1+\exp\frac{{\text{C}}_{\text{T}2}\cdot (\text{T}-{\text{T}}_{\text{M}})}{\text{R}\cdot {\text{T}}_{\text{s}}\cdot \text{T}}}$$

Where, C_T1_: constant, 95000 J·mol^−1^; C_T2_: constant, 230000 J·mol^−1^; T_M_: 314K; R: ideal gas constant, 8.314 J·K^−1^·mol^−1^; T: actual leaf temperature during sampling, K; T_s_: leaf temperature under standard conditions, 303 K.

### 2.6 Statistical analysis

All statistical analyses were performed using R software (version 4.5.0). Prior to statistical analyses, data normality and homogeneity of variance were evaluated using the Shapiro-Wilk test and Levene’s test, respectively. When necessary, logarithmic transformation was applied to improve normality and variance homogeneity. Differences in BVOC emission rates among tree species and seasons were analyzed using one-way analysis of variance (ANOVA), followed by Tukey’s honestly significant difference (HSD) test for multiple comparisons. Statistical significance was determined at (*P* < 0.05). Spearman correlation analysis was used to evaluate relationships between BVOC emission rates and environmental variables, including air temperature and PAR. Relationships between BVOC emissions and environmental factors were visualized using fitted trend lines.

## 3 Results and analysis

### 3.1 Differences in BVOCs emitted by different tree species

**Figure 3:**
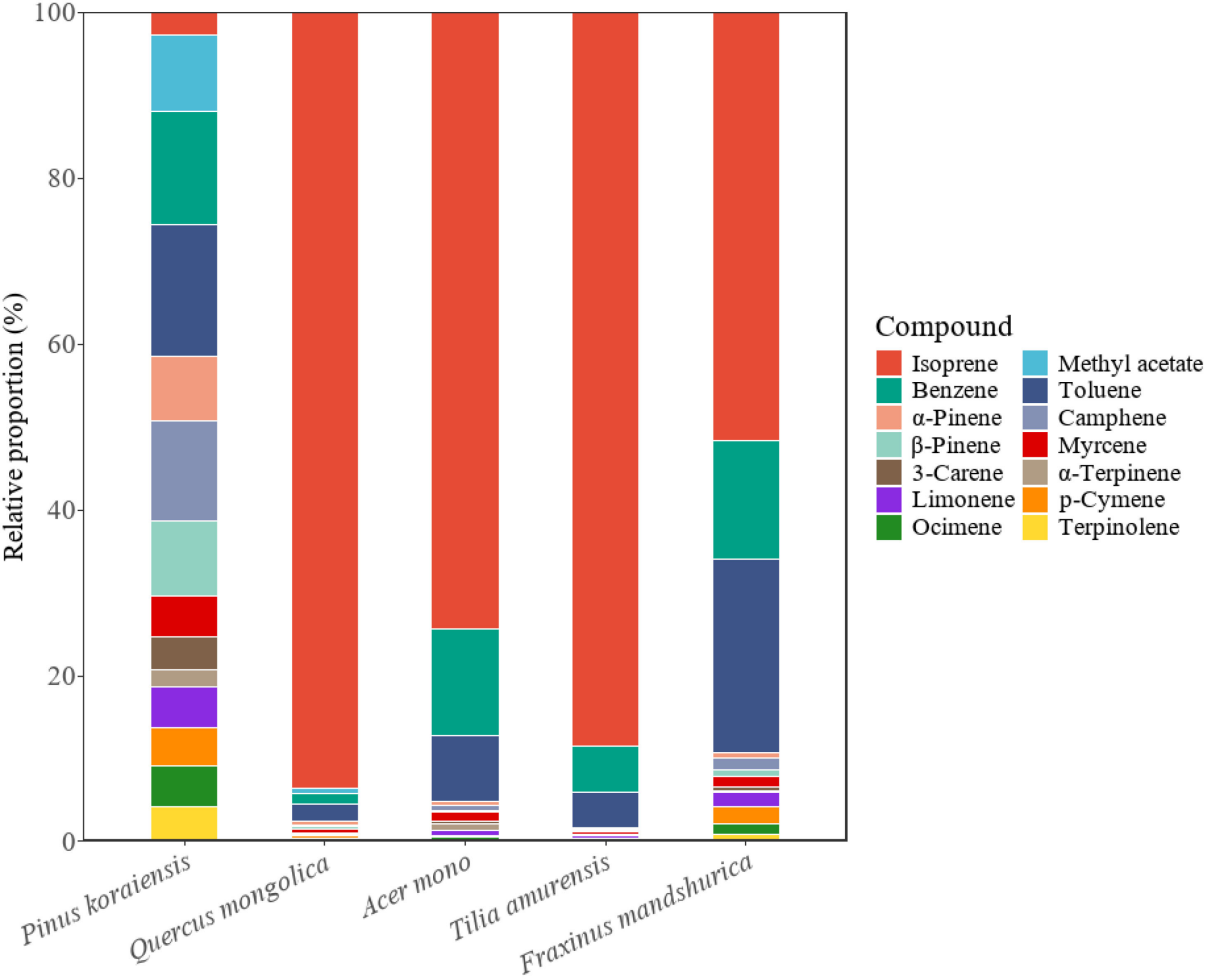
Composition of BVOC emissions from dominant tree species.

Clear interspecific differences in BVOC composition were observed among the investigated tree species (**Figure 3**). *Pinus koraiensis* was characterized by monoterpene-dominated emissions, with monoterpenes accounting for 58.5% of total BVOC emissions, whereas isoprene represented only a minor fraction (2.7%). Camphene and pinene compounds were the dominant monoterpenes in this species.

In contrast, all four broad-leaved tree species were primarily dominated by isoprene emissions. *Quercus mongolica* exhibited the highest isoprene proportion (93.6%), while *Tilia amurensis* also showed strong isoprene dominance (88.4%). *Acer mono* displayed a relatively lower isoprene proportion (74.3%) compared with the other broad-leaved species, and low but detectable aromatic compounds, mainly benzene and toluene, were also identified. *Fraxinus mandshurica* showed a transitional BVOC composition pattern in which isoprene remained the dominant component (51.7%), accompanied by detectable aromatic compounds.

Monoterpene composition also differed substantially among tree species. *Pinus koraiensis* was mainly characterized by camphene and pinene compounds, whereas broad-leaved species exhibited distinct dominant monoterpenes, including myrcene, limonene, ocimene, and α-terpinene. BVOC composition exhibited pronounced species specificity among the investigated tree species.

### 3.2 Emission rates differences of BVOCs among tree species

**Figure 4:**
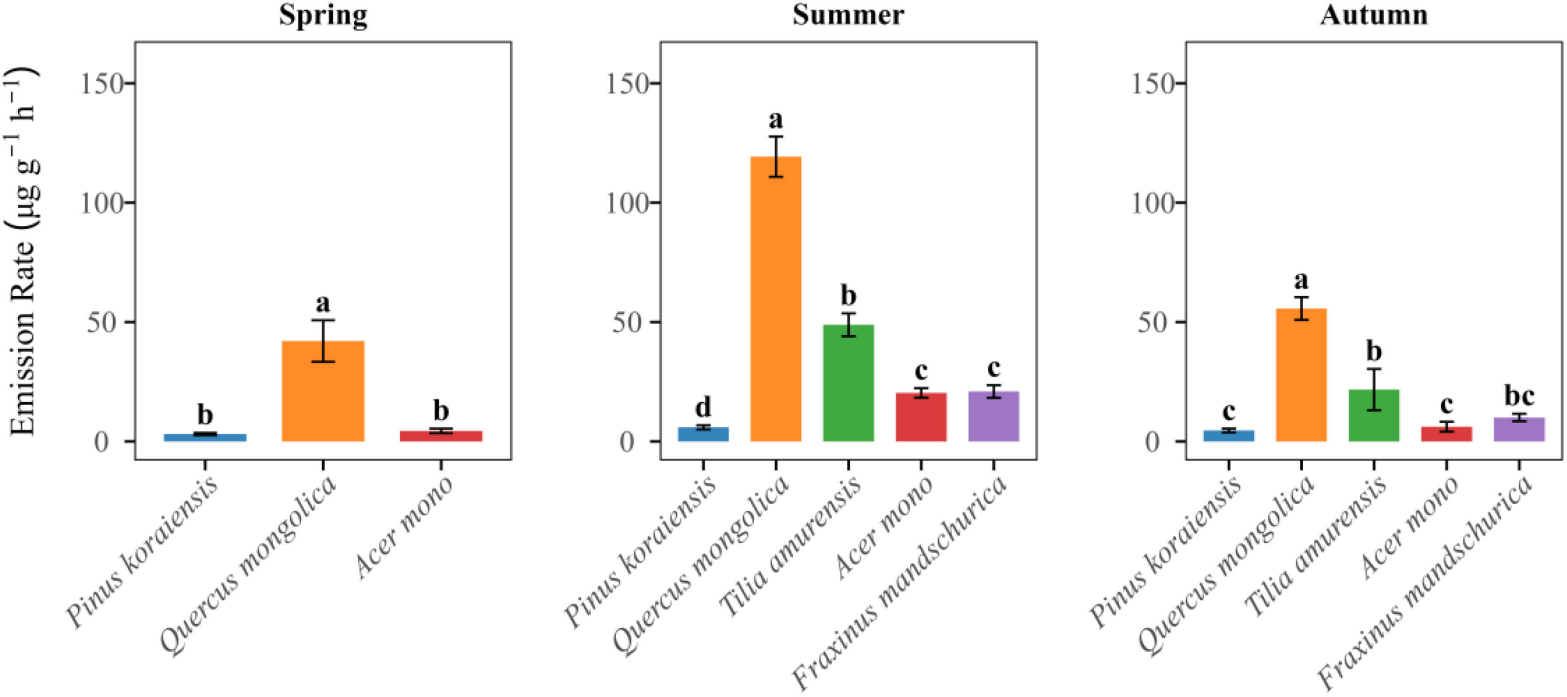
Total BVOC emission rates of different tree species. Values shown are raw measured emission rates under field conditions. Error bars represent standard errors. Different lowercase letters indicate significant differences among tree species within the same season (Tukey’s HSD test, *P* < 0.05). *Tilia amurensis* and *Fraxinus mandshurica* were not included in spring measurements because canopy leaf expansion was incomplete during the sampling period.

BVOC emission rates varied substantially among tree species and seasons (**Figure 4**). Overall, broad-leaved tree species exhibited higher BVOC emission rates than *Pinus koraiensis* across all observation periods. Among the investigated species, *Quercus mongolica* consistently showed the highest emission rates, whereas *Pinus koraiensis* maintained relatively low emission levels throughout the growing season. Seasonal variation in BVOC emissions was pronounced. In spring, *Quercus mongolica* exhibited significantly higher emission rates (42.06 ± 15.08 μg g^−1^ h^−1^) than *Pinus koraiensis* (3.10 ± 0.83 μg g^−1^ h^−1^) and *Acer mono* (4.41 ± 1.66 μg g^−1^ h^−1^). *Tilia amurensis* and *Fraxinus mandshurica* were not included in spring measurements because canopy leaf expansion had not yet been fully completed during the sampling period. In summer, BVOC emissions increased substantially in all broad-leaved species, and interspecific differences became more apparent. *Quercus mongolica* showed the highest emission rate (119.28 ± 26.65 μg g^−1^ h^−1^), followed by *Tilia amurensis* (48.85 ± 15.17 μg g^−1^ h^−1^). Emission rates of *Acer mono* and *Fraxinus mandshurica* remained significantly lower than those of *Quercus mongolica* and *Tilia amurensis*, while *Pinus koraiensis* maintained the lowest emission rate among all investigated species. In autumn, BVOC emission rates decreased in most species compared with summer values. Nevertheless, *Quercus mongolica* still maintained significantly higher emission rates (55.66 ± 11.68 μg g^−1^ h^−1^) than the other species. Tilia amurensis exhibited intermediate emission levels, whereas *Pinus koraiensis* and *Acer mono* showed relatively low emission rates. *Fraxinus mandshurica* did not differ significantly from either the intermediate- or low-emission groups.

**Figure 5:**
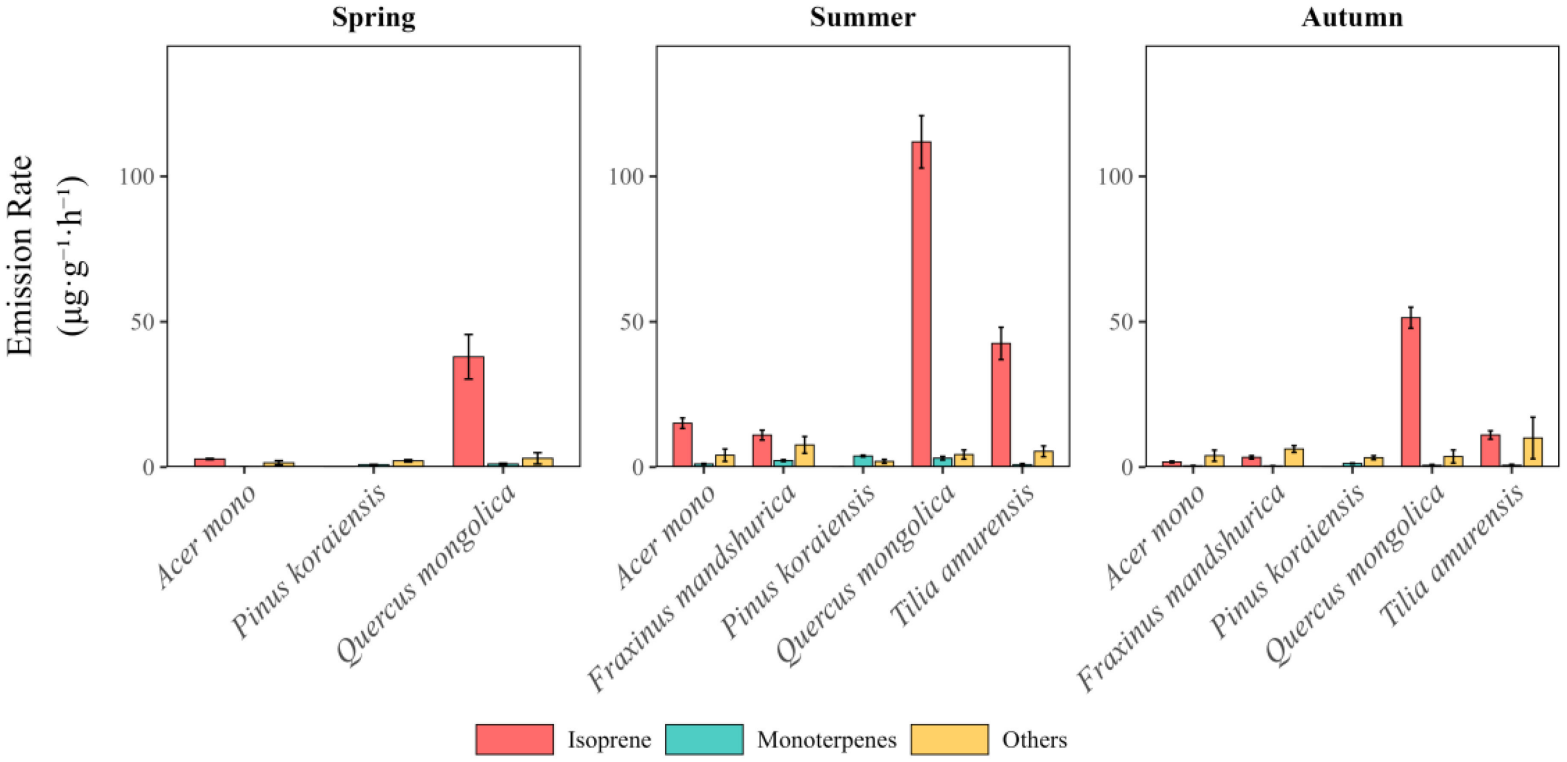
Emission rates of different BVOC components from various tree species. Values shown are raw measured emission rates under field conditions. “Others” includes methyl acetate and several minor BVOC compounds. Error bars represent standard errors. *Tilia amurensis* and *Fraxinus mandshurica* were not included in spring measurements because canopy leaf expansion was incomplete during the sampling period.

BVOCs were grouped into isoprene, monoterpenes, and other compounds according to chemical composition (**Figure 5**). Distinct species-specific emission patterns were consistently observed across seasons. *Pinus koraiensis* was characterized by relatively low isoprene emissions and comparatively higher monoterpene emissions throughout the observation period. In contrast, all four broad-leaved tree species were dominated by isoprene emissions, with *Quercus mongolica* showing the strongest isoprene dominance.

In spring, isoprene emission rates of *Quercus mongolica* were substantially higher than those of Acer mono and *Pinus koraiensis*. Emission rates of monoterpenes and other compounds remained comparatively low in all measured species during this period. In summer, interspecific differences in BVOC component emissions became more apparent. *Quercus mongolica* exhibited the highest isoprene emission rate, while Tilia amurensis also showed relatively high isoprene emissions compared with the other species. Monoterpene emissions remained relatively high in *Pinus koraiensis* but were comparatively low in the broad-leaved species. In autumn, isoprene emissions of *Quercus mongolica* decreased compared with summer values but still remained higher than those of the other species. *Pinus koraiensis* continued to exhibit relatively higher monoterpene emissions, whereas the remaining broad-leaved species showed comparatively low monoterpene emission levels.

**Figure 6:**
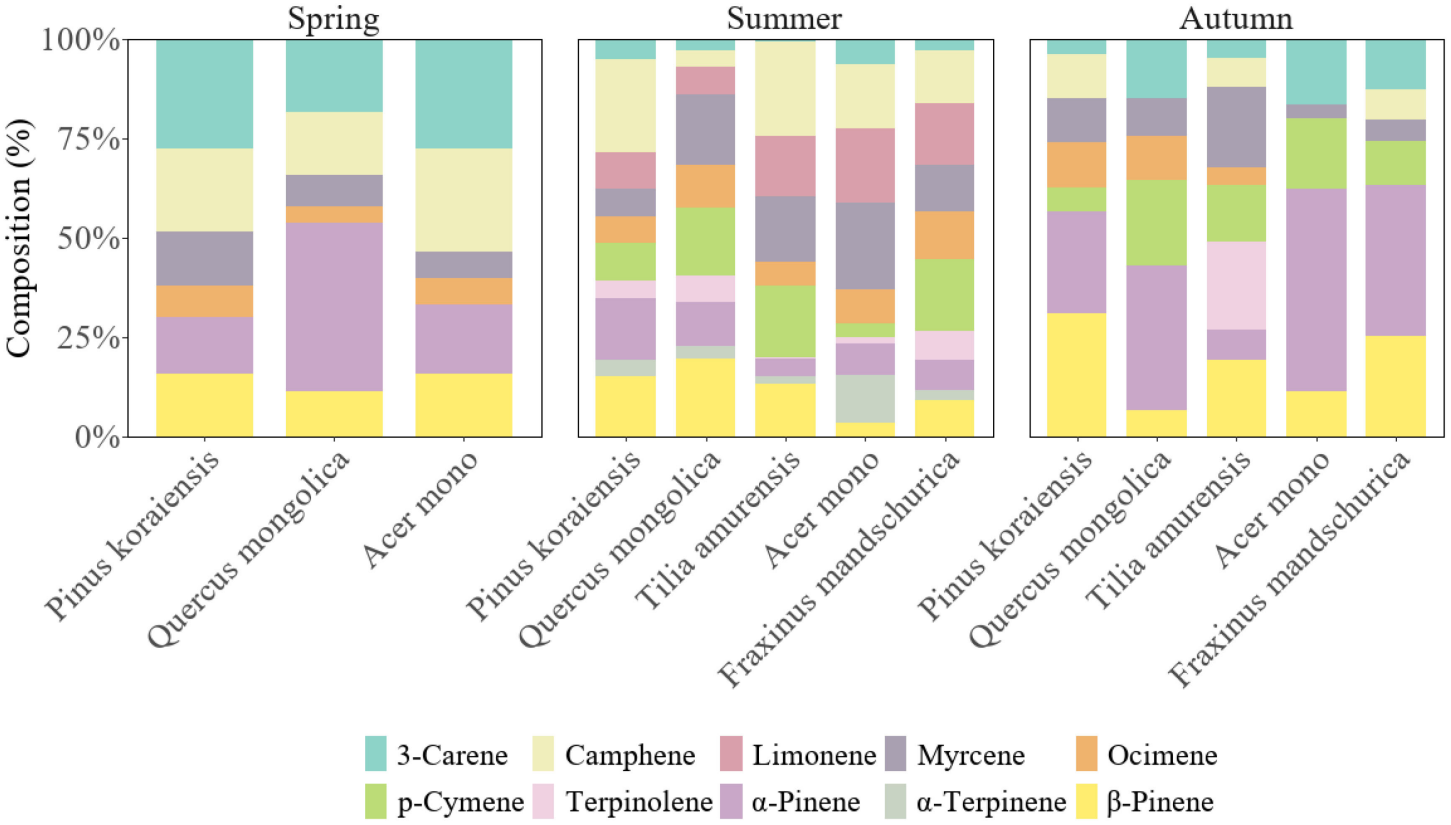
Relative composition of monoterpene compounds among dominant tree species. Different colors represent different monoterpene compounds, and the stacked bars indicate the relative proportion (%) of each compound within the total monoterpene composition.

Monoterpene composition differed substantially among tree species and seasons (**Figure 6**), and clear species-specific patterns were consistently observed throughout the observation period.

In spring, monoterpene emissions of *Pinus koraiensis* and *Acer mono* were mainly composed of 3-carene, camphene, and pinene compounds, whereas *Quercus mongolica* was strongly dominated by α-pinene and β-pinene. Distinct interspecific differences in monoterpene composition were therefore already apparent during the early growing season.

In summer, monoterpene composition diverged further among the investigated species. *Pinus koraiensis* exhibited relatively balanced proportions of camphene, α-pinene, and β-pinene, while *Quercus mongolica* was characterized by relatively high proportions of β-pinene, myrcene, and p-cymene. Acer mono was mainly dominated by myrcene and limonene, whereas *Fraxinus mandshurica* showed comparatively high proportions of p-cymene and limonene. Tilia amurensis exhibited a more complex monoterpene composition characterized by relatively high proportions of camphene, p-cymene, and myrcene.

In autumn, species-specific differences in monoterpene composition remained evident. *Pinus koraiensis* continued to be dominated by pinene compounds, whereas *Quercus mongolica* maintained relatively high proportions of α-pinene. *Acer mono* showed a marked increase in α-pinene proportion, resulting in strong α-pinene dominance during autumn. *Fraxinus mandshurica* was also dominated by α-pinene and β-pinene, while *Tilia amurensis* exhibited relatively high proportions of terpinolene, myrcene, and β-pinene.

### 3.3 Diurnal variation in BVOC emissions of dominant tree species

**Figure 7:**
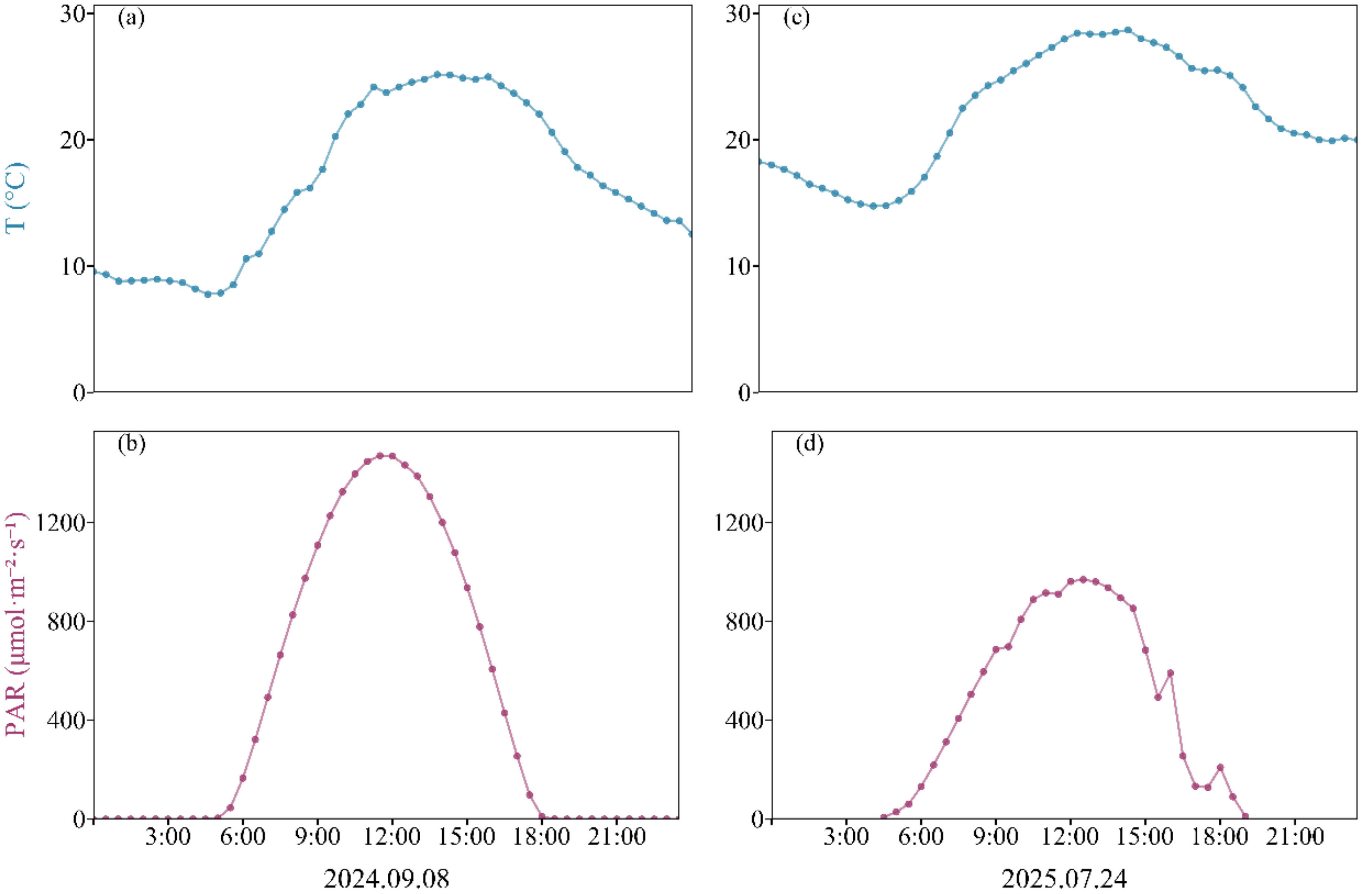
Diurnal variations in air temperature and PAR during representative observation periods. Panels **(a, c)** show air temperature variations, whereas panels **(b, d)** show PAR variations.

Diurnal variation analyses were conducted under relatively stable weather conditions during representative observation periods. Air temperature and PAR exhibited clear daytime variation patterns, with both variables generally increasing after sunrise and reaching peak values around midday or early afternoon (**Figure 7**).

#### 3.3.1 Diurnal variation in isoprene emission rates

**Figure 8:**
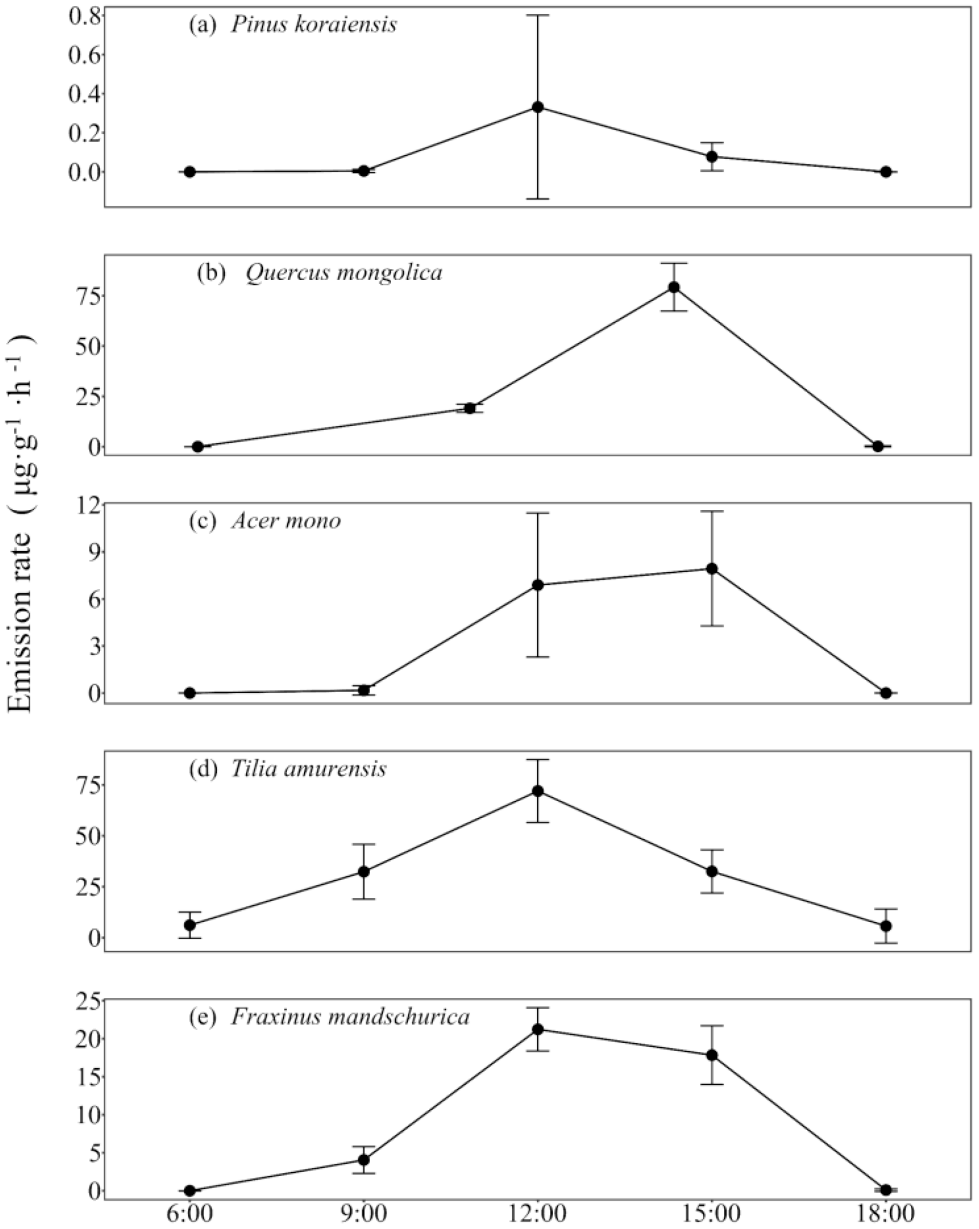
Diurnal variation of isoprene emission rates from dominant tree species **(a–e)**. Values shown are raw measured emission rates under field conditions. Error bars represent standard deviation.

Distinct interspecific differences in diurnal isoprene emission patterns were observed among the investigated tree species (**Figure 8**). Most species exhibited unimodal diurnal variation patterns, with peak emission rates generally occurring around midday or early afternoon.

*Pinus koraiensis* exhibited consistently low isoprene emission rates throughout the observation period, with a maximum daytime emission rate of only 0.33 ± 0.47 μg g^−1^ h^−1^. Emissions remained close to zero during the early morning and evening periods.

*Quercus mongolica* exhibited the highest isoprene emission rates among all investigated species and showed a pronounced unimodal diurnal pattern, with a midday peak of 79.27 ± 11.87 μg g^−1^ h^−1^. *Tilia amurensis* also exhibited relatively high isoprene emission rates, reaching a peak value of approximately 72.00 ± 15.45 μg g^−1^ h^−1^ around midday. Compared with *Quercus mongolica*, *Tilia amurensis* showed a broader daytime emission pattern, with detectable emissions already present during the morning period. *Acer mono* and *Fraxinus mandshurica* exhibited comparatively lower isoprene emission rates than *Quercus mongolica* and *Tilia amurensis*. In both species, isoprene emissions increased rapidly during midday and decreased substantially during the late afternoon and evening periods.

#### 3.3.2 Diurnal variation in monoterpene emission rates

**Figure 9:**
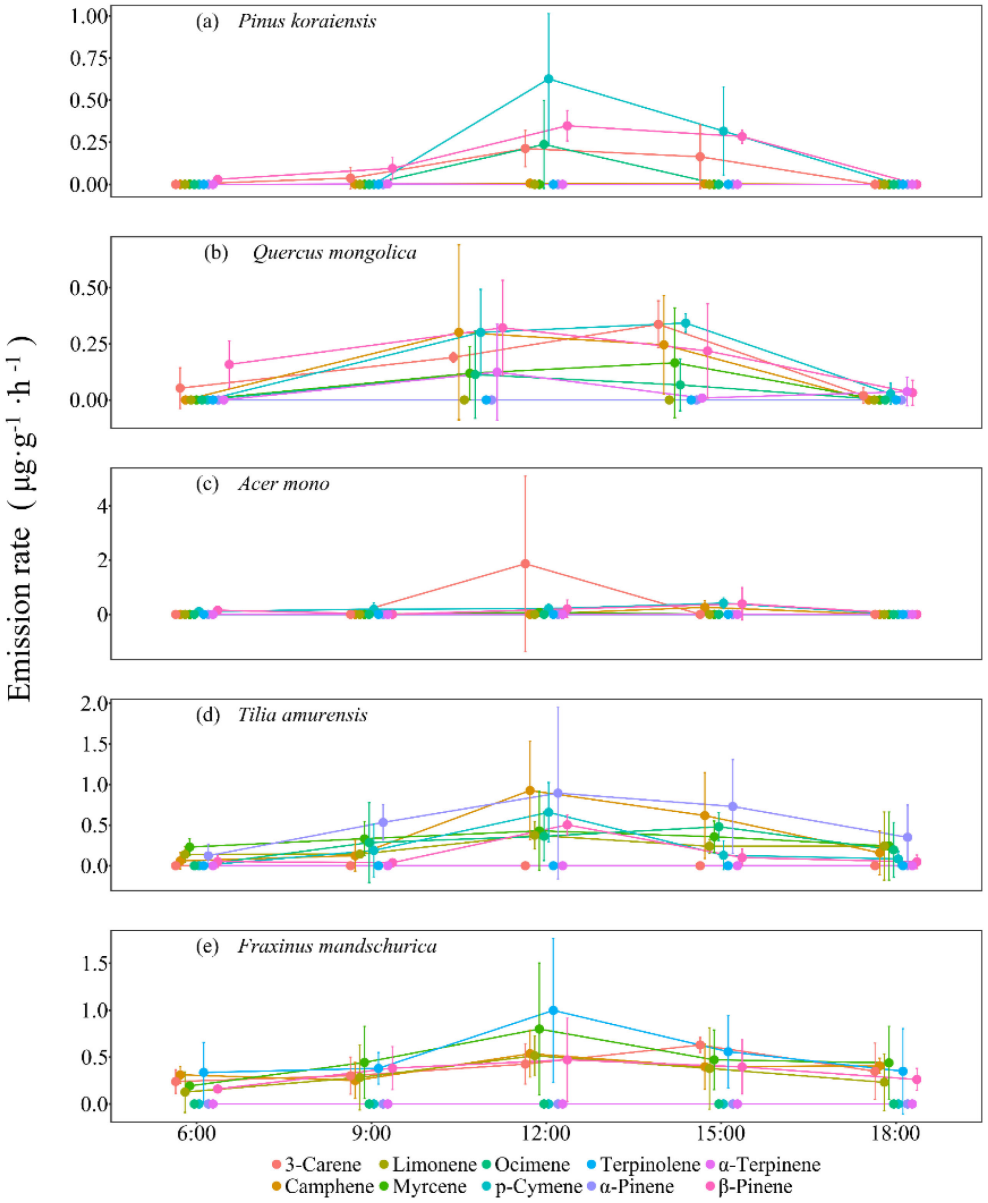
Diurnal variation of monoterpene emission rates from dominant tree species **(a–e)**. Values shown are raw measured emission rates under field conditions. Error bars represent standard deviation.

Monoterpene emission rates were generally much lower than isoprene emission rates across all investigated tree species (**Figure 9**). Compared with isoprene, monoterpene emissions exhibited broader and less pronounced diurnal variation patterns, with clear interspecific differences in dominant compounds and emission intensity.

*Pinus koraiensis* was characterized by relatively higher emissions of 3-carene, p-cymene, and β-pinene, with emission peaks generally occurring around midday. *Quercus mongolica* exhibited comparatively diverse monoterpene emissions, mainly dominated by β-pinene, p-cymene, and camphene, and showed daytime variation patterns similar to those observed for isoprene emissions. *Acer mono* exhibited relatively low monoterpene emission rates throughout the observation period, with only minor emissions of several monoterpene compounds detected during midday. *Tilia amurensis* showed relatively elevated emissions of α-pinene and camphene, with peak values generally occurring during the afternoon period. *Fraxinus mandshurica* exhibited comparatively diverse monoterpene compounds and maintained detectable emission levels during the late afternoon period after reaching midday emission maxima.

### 3.4 Seasonal differences in BVOC emission rates of dominant tree species

**Figure 10:**
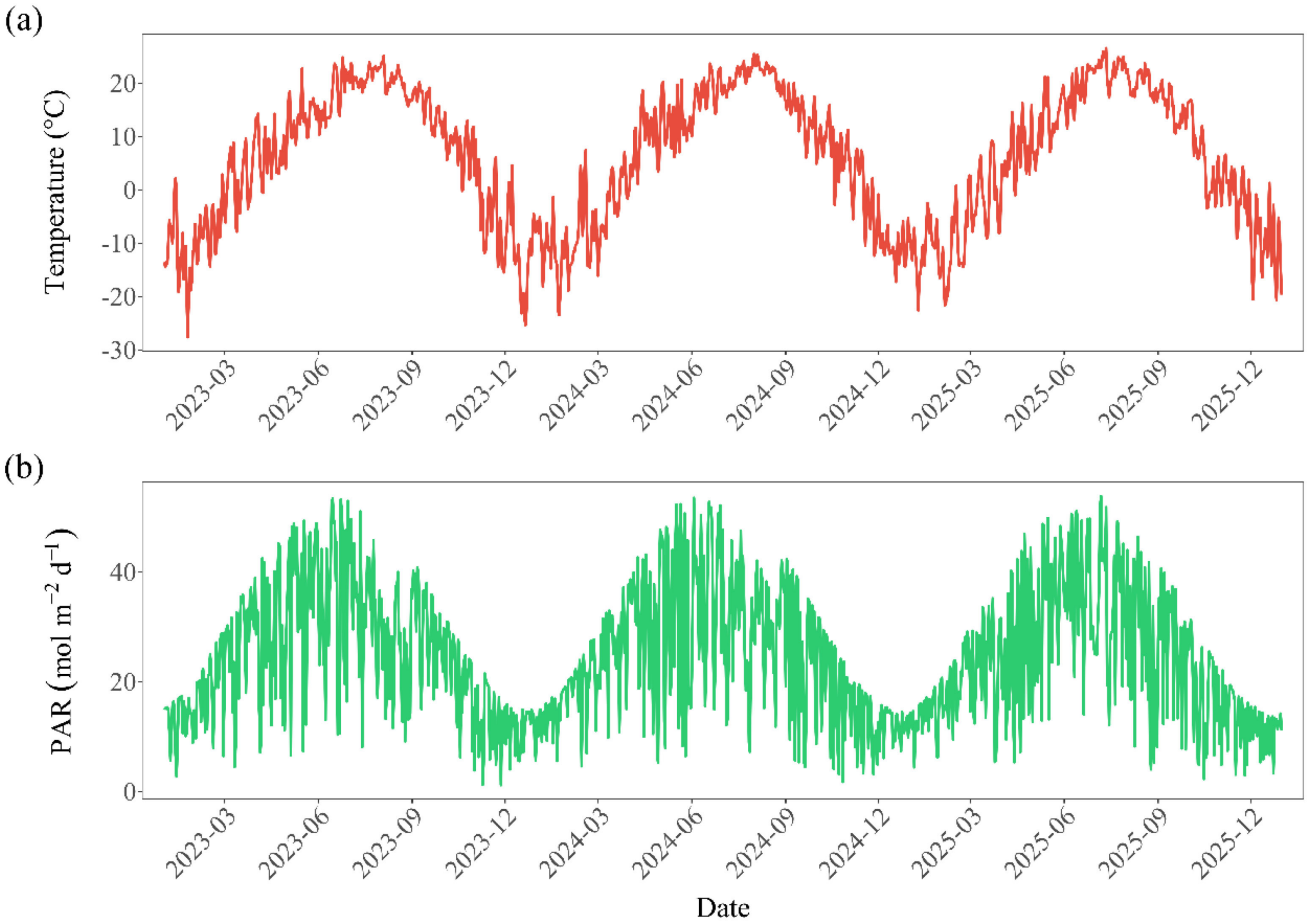
Seasonal variations in air temperature **(a)** and PAR **(b)** during the observation period.

Seasonal variations in air temperature and PAR during the observation period are shown in **Figure 10**. Both air temperature and PAR exhibited clear seasonal differences, with the highest values occurring during summer.

**Figure 11:**
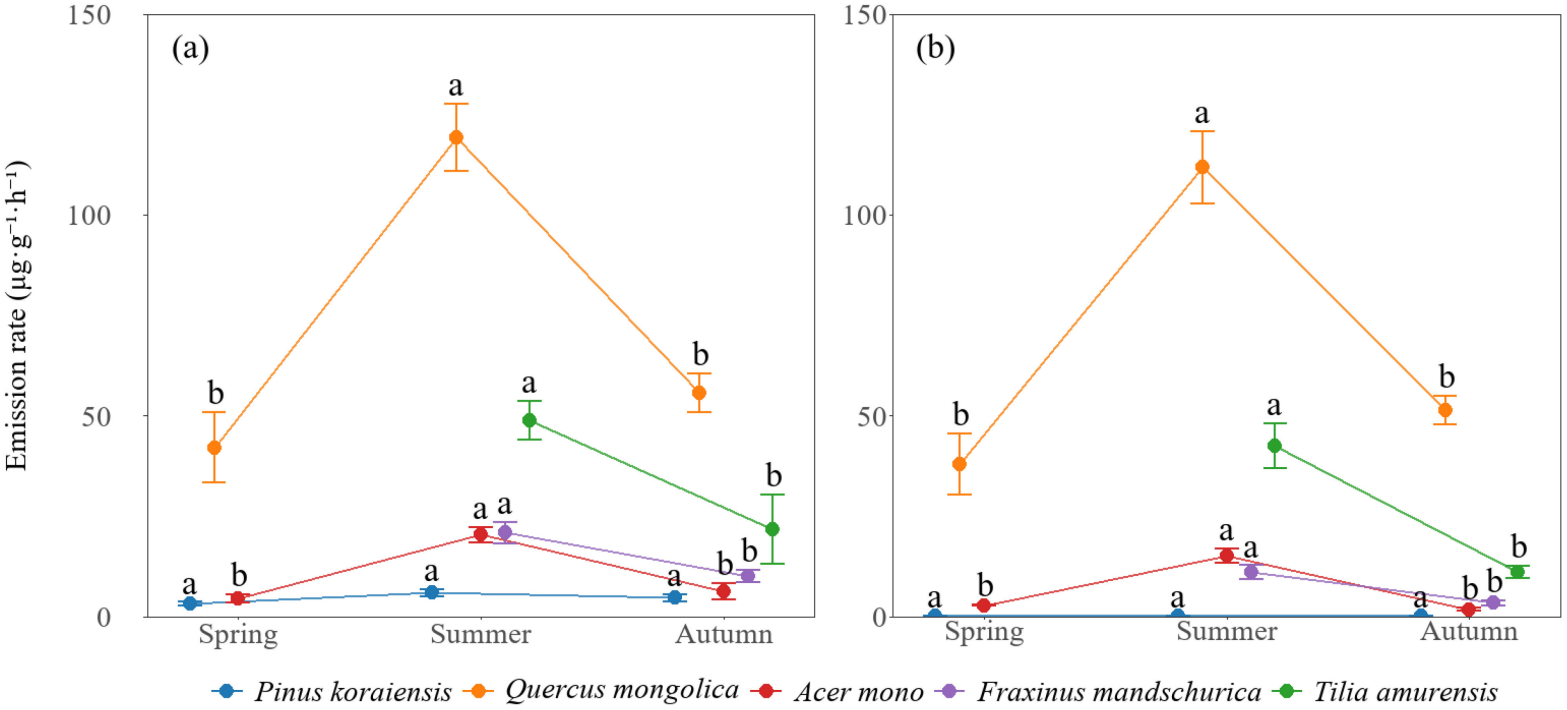
Seasonal variations in total BVOC and isoprene emission rates among dominant tree species. Values shown are raw measured emission rates under field conditions. Panel **(a)** shows total BVOC emission rates, and panel **(b)** shows isoprene emission rates. Error bars represent standard errors. Different lowercase letters indicate significant differences among seasons within the same tree species (Tukey’s HSD test, *P* < 0.05).

Total BVOC and isoprene emissions of the investigated tree species exhibited clear seasonal variation patterns (**Figure 11**). *Pinus koraiensis* maintained consistently low BVOC and isoprene emission levels throughout the observation period, with no significant seasonal differences.

In contrast, all four broad-leaved tree species exhibited pronounced summer peaks in BVOC emissions. Among them, *Quercus mongolica* showed the strongest seasonal variation, with substantially elevated emission rates during summer compared with spring and autumn. Similar seasonal patterns were also observed in *Acer mono*, *Tilia amurensis*, and *Fraxinus mandshurica*, all of which exhibited significantly higher BVOC emission rates in summer. Seasonal variation in these broad-leaved species was primarily associated with changes in isoprene emissions.

### 3.5 Effects of meteorological factors on BVOC emission rates

Correlation analyses were performed using raw measured BVOC emission rates under field conditions. BVOC emission rates exhibited clear species-specific relationships with environmental variables. In general, stronger positive correlations with temperature were observed than with PAR, although response patterns differed substantially among tree species and BVOC categories.

**Figure 12:**
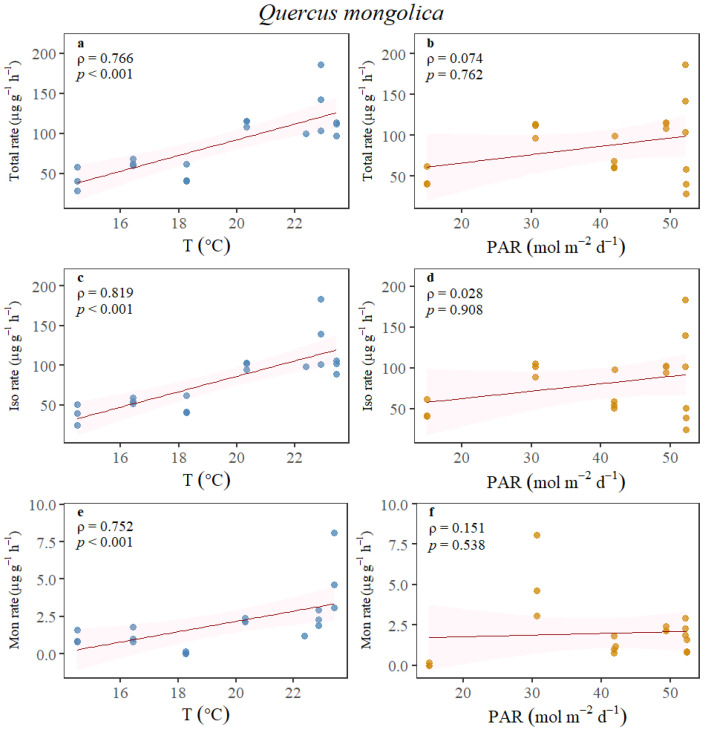
Correlation analysis of temperature **(a, c, e)**, PAR **(b, d, f)** and monthly BVOC emission rates of *Quercus mongolica.*.

**Figure 13:**
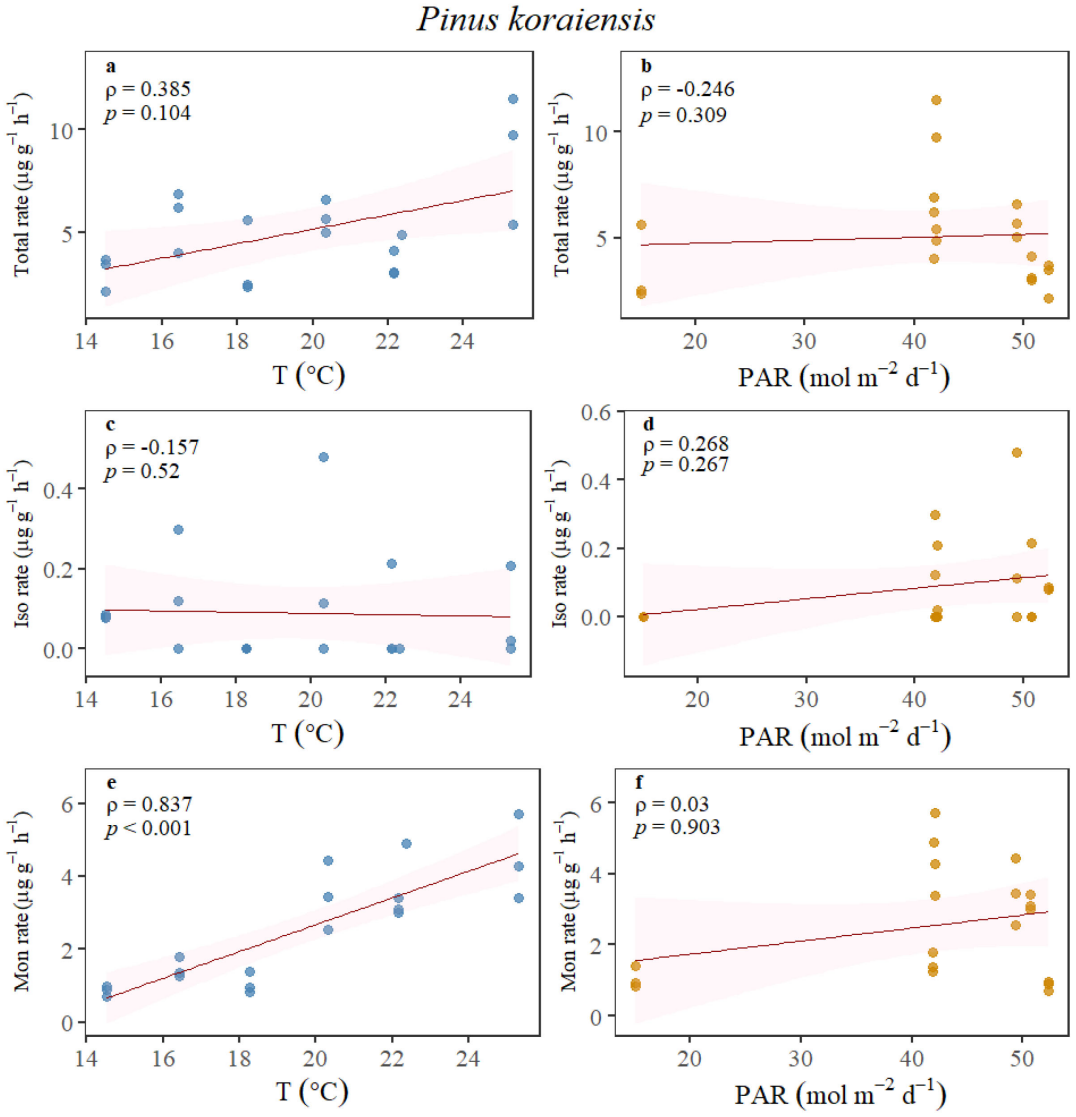
Correlation analysis of temperature **(a, c, e)**, PAR **(b, d, f)** and monthly BVOC emission rates of *Pinus koraiensis.*.

Among the investigated species, *Quercus mongolica* exhibited the strongest temperature-related response patterns, with significant positive correlations observed between temperature and total BVOC, isoprene, and monoterpene emissions (ρ=0.752-0.819, *P* < 0.001; **Figure 12**). In contrast, correlations with PAR were generally weak and not significant. Similar temperature-dominated relationships were also observed in *Pinus koraiensis*, in which monoterpene emissions showed a strong positive correlation with temperature, whereas isoprene emissions remained weak and showed no significant relationships with either environmental factor (**Figure 13**).

**Figure 14:**
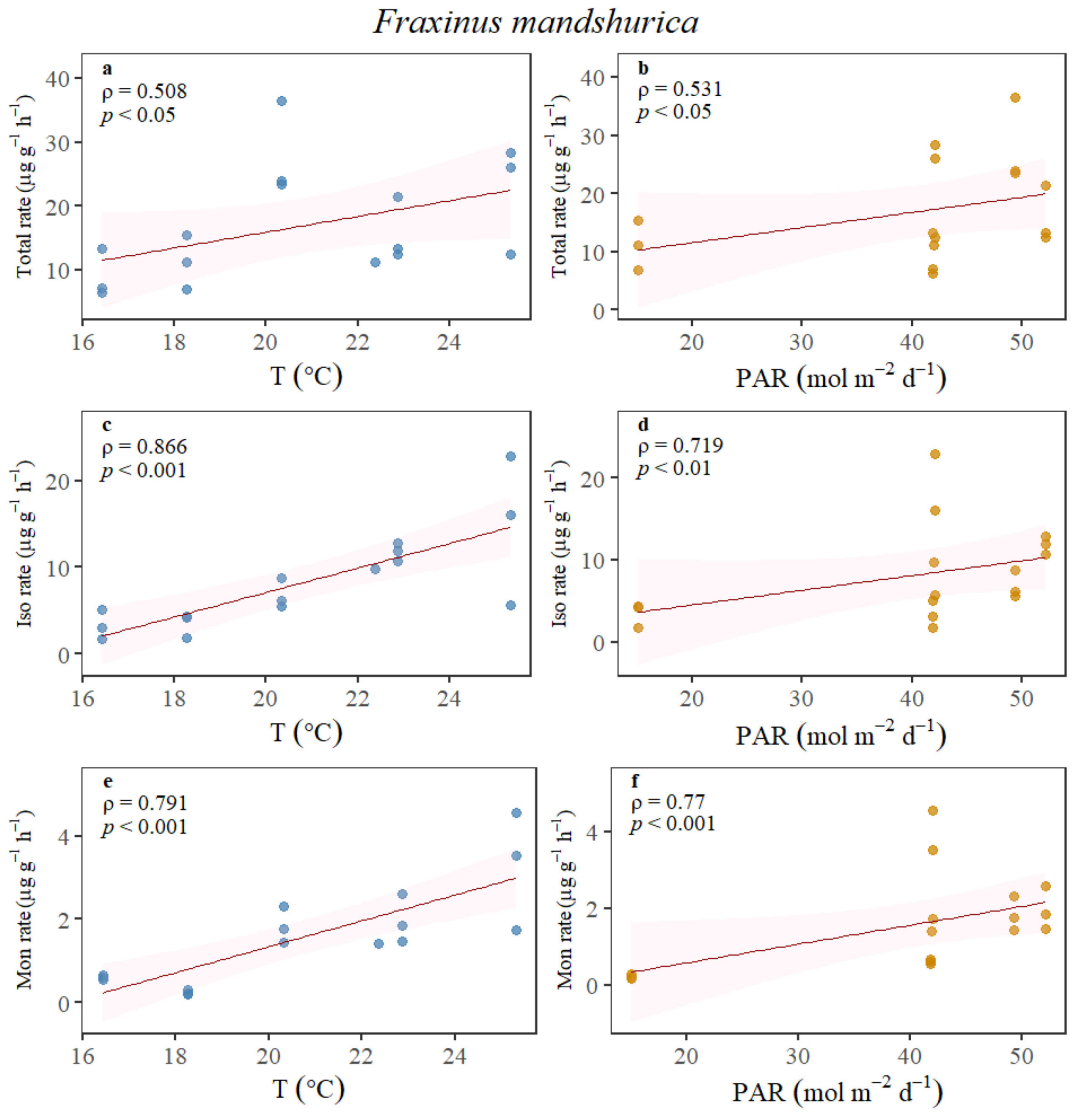
Correlation analysis of temperature **(a, c, e)**, PAR **(b, d, f)** and monthly BVOC emission rates of *Fraxinus mandschurica*.

**Figure 15:**
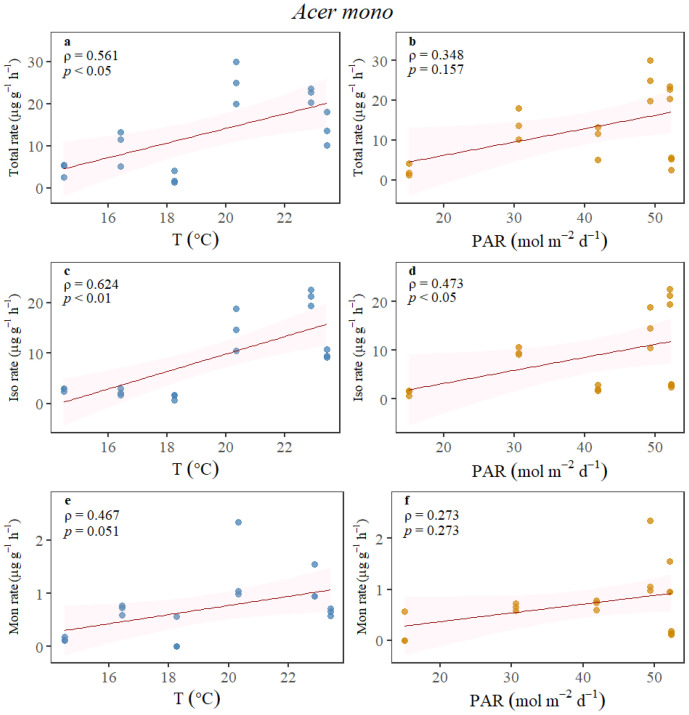
Correlation analysis of temperature **(a, c, e)**, PAR **(b, d, f)** and monthly BVOC emission rates of *Acer mono*.

**Figure 16:**
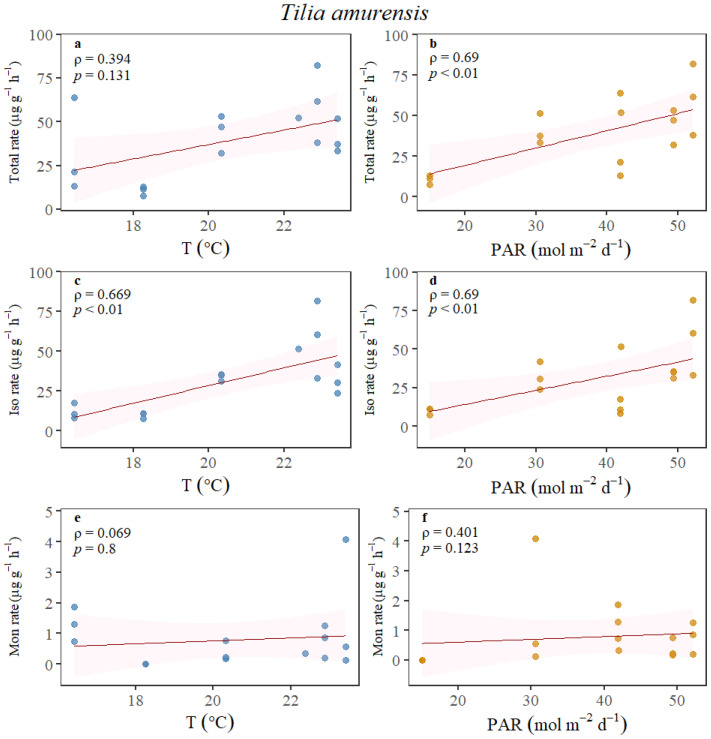
Correlation analysis of temperature **(a, c, e)**, PAR **(b, d, f)** and monthly BVOC emission rates of *Tilia amurensis*.

*Fraxinus mandshurica* showed significant positive correlations between BVOC emissions and both temperature and PAR, particularly for isoprene and monoterpene emissions (**Figure 14**). *Acer mono* also exhibited significant positive correlations between temperature and both total BVOC and isoprene emissions, whereas monoterpene emissions showed weak and non-significant relationships with environmental variables (**Figure 15**). In *Tilia amurensis*, significant positive correlations were mainly observed between isoprene emissions and both temperature and PAR, while monoterpene emissions showed no significant relationships with either environmental factor (**Figure 16**).

Overall, temperature appeared to be more closely associated with BVOC emission variation than PAR in the Changbai Mountain mixed forest ecosystem.

### 3.6 Standard emission factors

Standard BVOC emission factors of the dominant tree species were calculated using the G93 algorithm based on field-measured emission rates and corresponding environmental conditions during sampling. Clear interspecific differences in standardized emission factors were observed among the investigated species (**Table 2**).

**Table 2:** Standard emission factors of dominant tree species in the broad-leaved Korean pine forest in Northeast China.

Tree species	*Pinus koraiensis*	*Quercus mongolica*	*Tilia amurensis*	*Acer mono*	*Fraxinus mandshurica*
Isoprene	0.24	178.98	76.06	19.59	14.43
Methyl acetate	0.83	1.15	0.00	0.00	0.00
Benzene	1.24	2.24	4.86	3.40	3.98
Toluene	1.43	4.20	3.67	2.10	6.54
α-Pinene	0.71	0.64	0.07	0.13	0.16
Camphene	1.08	0.29	0.14	0.16	0.40
β-Pinene	0.82	0.75	0.16	0.05	0.20
Myrcene	0.44	0.85	0.34	0.26	0.39
3-Carene	0.37	0.33	0.04	0.09	0.09
α-Terpinene	0.18	0.14	0.12	0.20	0.08
Limonene	0.44	0.28	0.20	0.18	0.49
Cymene	0.41	0.53	0.23	0.04	0.55
Ocimene	0.45	0.39	0.12	0.12	0.36
Terpinene	0.38	0.35	0.02	0.03	0.24

Units are μg·g^−1^·h^−1^.

*Quercus mongolica* exhibited the highest standardized BVOC and isoprene emission factors among all investigated tree species, whereas *Pinus koraiensis* maintained consistently low standardized isoprene emission factors but comparatively higher monoterpene emission potentials. *Tilia amurensis* and *Fraxinus mandshurica* showed intermediate standardized emission levels, while *Acer mono* exhibited relatively lower standardized BVOC emission factors compared with the other broad-leaved species.

## 4 Discussion

### 4.1 Differences in BVOCs composition among tree species

The present study demonstrated pronounced species-specific differences in BVOC composition among dominant tree species in the broad-leaved Korean pine forest of Northeast China. A clear functional differentiation pattern was observed, with monoterpene-dominated emissions in the coniferous species *Pinus koraiensis* and isoprene-dominated emissions in the broad-leaved species. Similar compositional patterns have been widely reported in temperate forest ecosystems and are generally considered characteristic of functional differences between coniferous and broad-leaved tree species (Guenther et al., 1995; Sharkey and Yeh, 2001; Luo et al., 2025).

The dominance of monoterpene emissions in *Pinus koraiensis* is likely associated with the well-developed resin duct system commonly found in coniferous species. Resin ducts can store substantial amounts of monoterpenoids, allowing emissions to originate not only from *de novo* synthesis but also from volatilization of pre-existing storage pools (Duan et al., 2023). In the present study, α-pinene, β-pinene, and camphene were the dominant monoterpene compounds emitted by *Pinus koraiensis*, which is consistent with previous reports for coniferous species in temperate forests (Mermet et al., 2021; Bohlmann and Gershenzon, 2009; Degenhardt et al., 2009; Trapp and Croteau, 2001). Compared with broad-leaved species, this storage-based emission mechanism may also explain the relatively stable monoterpene emission patterns and weaker short-term environmental responses observed in *Pinus koraiensis*.

In contrast, the broad-leaved species investigated in this study were primarily dominated by isoprene emissions. *Quercus mongolica* exhibited particularly strong isoprene dominance, with isoprene accounting for more than 90% of total BVOC emissions, indicating that this species can be classified as a typical high-isoprene emitter. Previous studies have shown that isoprene emissions from broad-leaved species are closely linked to photosynthetic carbon metabolism (Moradi et al., 2025)and chloroplastic MEP-pathway activity (Chen et al., 2025). Because isoprene is mainly synthesized through rapid *de novo* pathways rather than long-term storage, its emissions are generally more sensitive to short-term environmental variation, particularly temperature and light conditions. This interpretation is also supported by the strong positive relationships between isoprene emissions and environmental variables observed in *Quercus mongolica* in the present study.

*Fraxinus mandshurica* displayed a mixed BVOC composition pattern characterized by both isoprene and detectable aromatic compounds, further indicating species-specific differences in BVOC composition among the investigated tree species. Although aromatic compounds such as benzene and toluene are commonly considered anthropogenic VOCs, previous studies have reported that plants may release trace aromatic compounds under specific physiological or environmental conditions, including oxidative stress and secondary metabolic processes (Heiden et al., 1999; Kesselmeier and Staudt, 1999; White et al., 2009; Misztal et al., 2015). In the present study, blank correction was applied during all emission-rate calculations to minimize potential contamination from ambient air and the sampling system.

Although monoterpenes accounted for a relatively small proportion of total BVOC emissions in most broad-leaved species, distinct species-specific differences in monoterpene composition were still observed. For example, Acer mono was characterized by relatively high proportions of myrcene and limonene, whereas *Tilia amurensis* showed comparatively high proportions of camphene, p-cymene, and myrcene. Similar species-specific monoterpene patterns have also been reported in previous studies (Zeng et al., 2024; Turlings and Erb, 2018; Peng et al., 2017; Dudareva et al., 2006), indicating relatively stable compositional characteristics among different tree species. These differences may reflect variations in terpene synthase activity, metabolic pathways, and ecological adaptation strategies among species.

In addition to interspecific variation, BVOC composition also exhibited clear seasonal dynamics. Several monoterpene compounds, including myrcene, ocimene, and p-cymene, showed substantial seasonal variation among tree species, indicating dynamic regulation of BVOC composition during different phenological stages and environmental conditions. Seasonal changes in temperature, radiation (Farré-Armengol et al., 2017), and physiological activity (Rodríguez-Flores et al., 2025)may jointly influence carbon allocation and volatile synthesis processes, thereby contributing to seasonal shifts in BVOC composition. However, the specific ecological and physiological functions of individual compounds were not directly investigated in the present study and therefore require further research.

### 4.2 Driving mechanisms of seasonal variation in BVOC emissions

The present study demonstrated pronounced seasonal variation in BVOC emissions, with most broad-leaved tree species exhibiting clear summer emission peaks. This seasonal pattern was generally consistent with the seasonal dynamics of temperature and PAR, suggesting that environmental conditions during summer strongly promote BVOC synthesis and release.

Among the investigated BVOC categories, isoprene exhibited the strongest seasonal response. Previous studies have shown that isoprene synthesis is closely coupled with photosynthetic metabolism and chloroplastic MEP-pathway activity, making its emissions highly sensitive to temperature and light conditions (Sharkey et al., 2007; Monson et al., 2012). Higher summer temperatures can enhance enzymatic activity, substrate availability, and volatile diffusion from leaf tissues to the atmosphere, thereby promoting elevated BVOC emission rates (Vettikkat et al., 2023). In the present study, *Quercus mongolica* and *Tilia amurensis* exhibited particularly strong summer increases in isoprene emissions, which was consistent with the strong positive relationships between isoprene emissions and temperature observed in the correlation analysis.

Compared with isoprene, monoterpene emissions generally exhibited weaker seasonal variation and more species-specific response patterns. In *Pinus koraiensis*, monoterpene emissions were likely associated not only with *de novo* synthesis but also with volatilization from storage pools within resin ducts (Chen et al., 2025). This storage-based emission mechanism may partly explain the relatively stable seasonal emission patterns and weaker short-term environmental responsiveness observed in *Pinus koraiensis* compared with the broad-leaved species. In contrast, monoterpene emissions from broad-leaved species appeared to be more closely associated with instantaneous environmental variation and physiological activity during the growing season.

PAR provides the energy basis for photosynthetic carbon assimilation and is closely linked to isoprene synthesis (Lyu et al., 2021; Zhang et al., 2026). However, compared with temperature, the influence of PAR was less pronounced in the present study. One possible explanation is that peak PAR and temperature conditions did not always occur synchronously during field observations, thereby weakening the apparent statistical relationship between PAR and BVOC emissions. In addition, the relatively limited sampling frequency under field conditions may also have reduced the detectability of independent PAR effects. Future studies combining controlled-environment experiments with continuous field observations may help further clarify the independent contributions of temperature and radiation to BVOC emission processes.

In addition, the standardization algorithm used in this study assumed that monoterpene emissions were primarily temperature-dependent. However, previous studies have shown that monoterpene emissions in some species may also be influenced by light conditions (Dindorf et al., 2006; Taipale et al., 2011). Therefore, the temperature-only algorithm applied here may introduce uncertainties for certain compounds and species. Although the investigated species in this study generally showed relatively weak PAR responses, future studies should further evaluate the applicability of algorithms incorporating both temperature and light effects under different species and environmental conditions.

Overall, the results indicate substantial interspecific differences in BVOC environmental responses among different plant functional types. Broad-leaved species generally exhibited stronger seasonal variability and environmental responsiveness than the evergreen conifer *Pinus koraiensis*. Such functional-type differentiation (Arneth et al., 2008)may contribute substantially to the spatiotemporal heterogeneity of BVOC emissions in temperate mixed forests (Sindelarova et al., 2014)and should be considered in future regional BVOC emission inventories and atmospheric chemistry models.

## 5 Conclusion

This study investigated the BVOC emission characteristics of five dominant tree species in the broad-leaved Korean pine forest of Northeast China and revealed pronounced species-specific and functional-type differences in BVOC composition and emission patterns.

*Pinus koraiensis* was dominated by monoterpene emissions, whereas the broad-leaved species were primarily dominated by isoprene emissions, with *Quercus mongolica* exhibiting the strongest isoprene emission characteristics. BVOC emissions showed clear seasonal and diurnal variation patterns, with generally higher emission rates during summer and stronger environmental responsiveness in broad-leaved species than in *Pinus koraiensis*.

Temperature showed stronger relationships with BVOC emissions than PAR, although environmental response patterns differed among tree species and BVOC categories. The observed emission dynamics were jointly influenced by environmental conditions, plant functional type, and physiological characteristics.

The present study provides field-based BVOC emission data and standardized emission factors for dominant tree species in the broad-leaved Korean pine forest of Northeast China, which may contribute to future regional BVOC emission inventories and atmospheric chemistry modeling.

## Data Availability

The datasets presented in this article are not readily available because the data are part of an ongoing study. Requests to access the datasets should be directed to peidingyi21@mails.ucas.ac.cn.
